# Influence of infrastructure, ecology, and underpass-dimensions on multi-year use of Standard Gauge Railway underpasses by mammals in Tsavo, Kenya

**DOI:** 10.1038/s41598-022-09555-5

**Published:** 2022-04-05

**Authors:** Fredrick Lala, Patrick I. Chiyo, Patrick Omondi, Benson Okita-Ouma, Erustus Kanga, Michael Koskei, Lydia Tiller, Aaron W. Morris, William J. Severud, Joseph K. Bump

**Affiliations:** 1 Wildlife Research and Training Institute, P.O. Box 842-20117, Naivasha, Kenya; 2 Department of Biology, Duke University, P.O. Box 90338, Durham, NC 27708 USA; 3 University of Minnesota, 2003 Upper Buford Circle, St. Paul, MN 55108-6074 USA; 4 Save the Elephants, P.O. Box 54667, Nairobi, 00200 Kenya; 5 Ministry of Tourism and Wildlife, P. O. Box 41394, Nairobi, 00100 Kenya

**Keywords:** Ecology, Zoology

## Abstract

Rail and road infrastructure is essential for economic growth and development but can cause a gradual loss in biodiversity and degradation of ecosystem function and services. We assessed the influence of underpass dimensions, fencing, proximity to water and roads, Normalized Difference Vegetation Index (NDVI), presence of other species and livestock on underpass use by large and medium-sized mammals. Results revealed hyenas and leopards used the underpasses more than expected whereas giraffes and antelopes used the underpasses less than expected. Generalized linear mixed-effects models revealed that underpass height influenced use by wildlife, with several species preferring to use taller underpasses. Electric fencing increased underpass use by funneling species towards underpasses, except for elephants and black-backed jackal for which it reduced underpass passage. We also found that the use of underpasses by livestock reduced the probability of use by nearly 50% for wildlife species. Carnivore species were more likely to cross underpasses used by their prey. Buffalo, livestock, and hyenas used underpasses characterized by vegetation with higher NDVI and near water sources while baboons, dik-diks and antelope avoided underpasses with high NDVI. Our findings suggest a need for diverse and comprehensive approaches for mitigating the negative impacts of rail on African wildlife.

## Introduction

Linear infrastructure is essential for economic growth and development^1^, but it is also widely regarded as a catalyst for biodiversity loss in natural ecosystems^2–7^. Moreover, an increase in infrastructure is predicted to occur mostly in the tropics where there are high levels of biodiversity and susceptible ecosystems^8,9^. Generally, railway traffic, like road traffic, may negatively impact biodiversity through increased wildlife mortality from road and rail kills, loss of population connectivity, habitat fragmentation, pollution (e.g., noise, chemical and light), and habitat loss^6,10–14^. Although railways may have similar impacts as roads, little attention has been paid towards mitigation of the effects of railways on wildlife. Perhaps, this is because the impacts of railways are perceived to be negligible due to a lack of awareness and reporting, lower traffic flow than roads, and long traffic-free intervals among others^15^. However, with increasing railway traffic volumes and the expansion of high-speed trains, wildlife mortality from railways will likely increase^10,16^, and this will demand implementation and evaluation of mitigation measures to reduce wildlife mortality.

Wildlife corridors (e.g., underpasses, overpasses, culverts) along highways traversing conservation areas are a valuable mitigation tool for enhancing the permeability of transportation infrastructure for wildlife while preventing wildlife mortalities from vehicle collisions and encouraging connectivity^17–20^. Several research studies, mostly in North America and Europe, have examined the effectiveness of wildlife underpasses along highways^19,21–30^, but limited studies exists for railways^18,31–33^. Moreover, there is a dearth of information on wildlife use of underpasses associated with roads or railways by wildlife in the African continent where these designs and technologies are increasingly being adopted (but see examples^34–36^).

North American and European studies reveal that the effectiveness of underpasses is dependent on design factors such as size dimensions (i.e., height, length, and width) and location^27,37–39^. In addition, ecological factors such as the presence of vegetation cover, forage, species involved, species interactions (e.g., predator–prey) and human activities are also important^26,33,38,40^. The optimal characteristics for wildlife underpasses along highways are known to be species-specific^28,41^, suggesting that it may be difficult to create universally optimal designs in areas with diverse wildlife species. This is further compounded by species and individuals differing in their propensity to use underpasses^42,43^. An understanding of how railway and road underpass design influences use by African savannah wildlife is lacking. There is an urgent need to understand how modern railway infrastructure is differentially impacting the connectivity and conservation of various African species because many rail infrastructure projects are planned or underway in Africa^44^. This is accomplished specifically by identifying species that are negatively impacted by railway development and may need alternative interventions, especially for species for which wildlife corridors traversing railways appear to have little to no positive influence.

The use of fencing to funnel species towards underpasses, so as to minimize collisions with automobiles on highways, has been demonstrated to be effective in Europe and North America^22,45,46^. While fencing is an effective method for funneling species, it might limit the migration of species with routine migratory routes if these are fenced, enhancing genetic isolation^47–49^. This highlights the need to understand how different African savannah wildlife species use wildlife corridors and the influence of fencing on their effectiveness. Moreover, because railways are frequently co‐aligned with roads, to form infrastructure corridors^12^, the impact of such parallel road and railways on the effectiveness of wildlife passages and fencing along highways is less known.

Here we examine for the first time in an African savannah ecosystem, the influence of a Standard Gauge Railway’s (SGR hereafter) underpass design (type, height, and width), proximity to roads, fencing, livestock, and associated ecological factors on the likelihood of crossing by large- and medium-sized mammals in the Tsavo Conservation Area (TCA), Kenya. Specifically, we address six key questions: (1) Are some species more likely than others to use underpasses? (2) Is fencing effective in funneling wildlife and livestock species towards underpasses? (3) Do factors such as the type of underpass (bridge or culvert), proximity of underpass to a paved road, and the dimensions (i.e., width and length) of the underpass, enhance or inhibit their use by different wildlife species? (4) Do ecological variables such as the proximity of underpass to perennial water sources, NDVI (green biomass index) around the underpass influence their use by different wildlife species? (5) Does the presence of livestock or wildlife predators along underpasses reduce the probability of their use by other non-carnivore wildlife species? (6) Does the presence of prey species influence the probability of carnivores using underpasses? Answers to these questions are key to addressing the knowledge gap that exists regarding impacts of rail on African savannah wildlife.

## Study area and methods

### Study area

**Figure 1:**
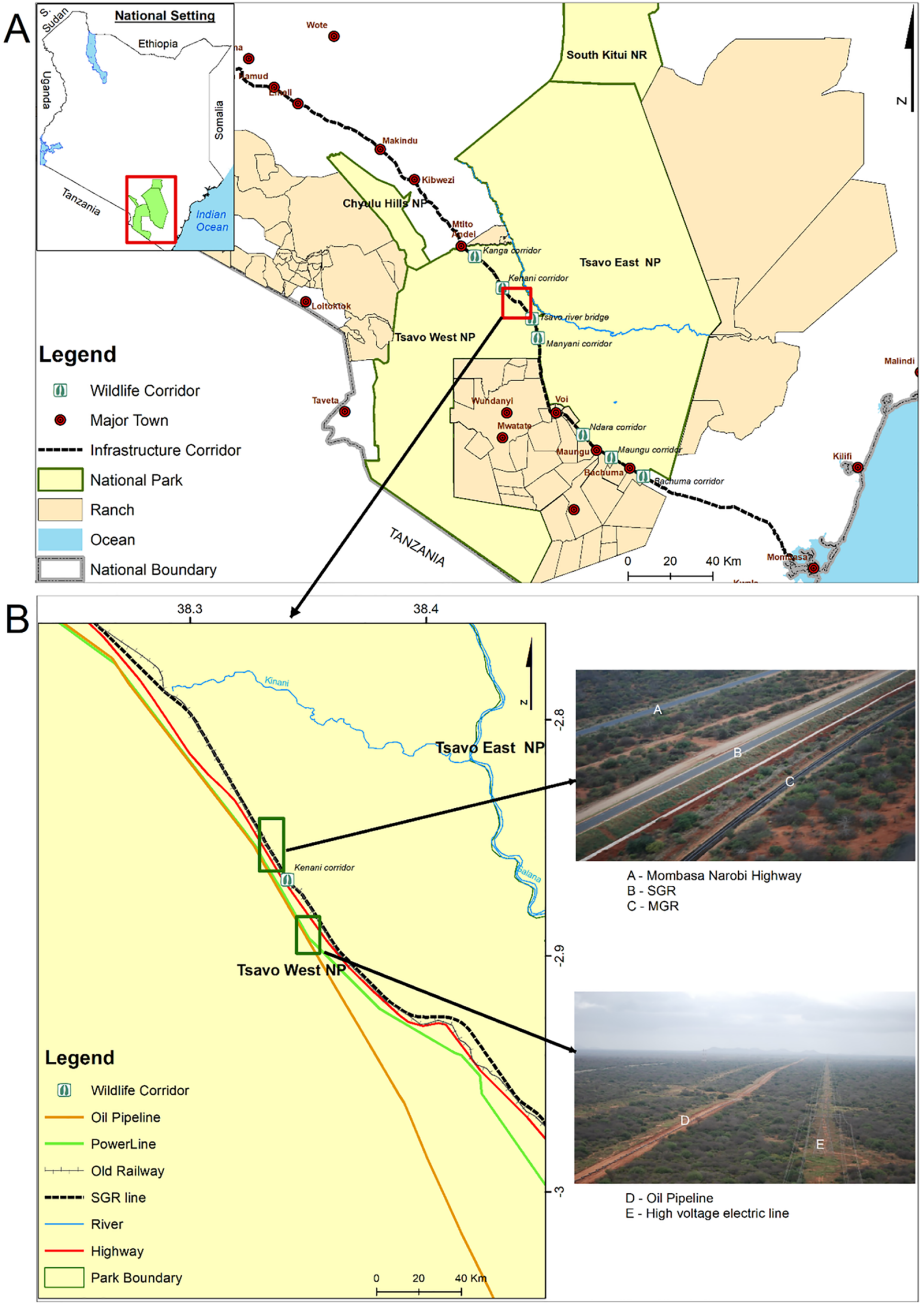
The Tsavo Conservation Area (TCA) in south-eastern Kenya (inset). The bold dashed black line indicates the Mombasa-Nairobi highway infrastructure corridor (MNHIC). adjacent to Tsavo National Parks (**A**) and the transport infrastructures that cuts through the Tsavo Conservation Area in southeastern Kenya (**B**). Maps were developed using ArcGIS Software version 10.2.2 (ESRI 2015).

The Tsavo Conservation Area hereafter referred to as TCA, lies in South-Eastern Kenya and covers an area of 42,000 km^2^ which includes three national parks, one game reserve, and several private ranches (Fig. 1a). The three national parks include Tsavo West National Park (~ 7000 km^2^), Tsavo East National Park (~ 14,000 km^2^), Chyulu Hills National Park (~ 700 km^2^), and South Kitui National Reserve (~ 1833 km^2^). The private ranches include Taita, Galana, Kulalu, adjacent private and communal lands^50^. The TCA is a tourism flagship of the Kenya Wildlife Service (KWS) and it generates nearly 50% of revenue for KWS^51^. The conservation area is home to the endangered savannah elephant (*Loxodonta africana*) maintaining approximately 40% of Kenya's population, as well as 18% of Kenya's population of the critically endangered black rhinoceros (*Diceros bicornis*). In addition, it is also the home to the critically endangered Hirola antelope (*Beatragus hunteri*), and the endangered Grevy’s zebra (*Equus grevyi*). Other herbivores in the TCA include the greater kudu (*Tragelaphus strepsiceros*), lesser kudu (*Tragelaphus imberbis*), Kirk's dik-dik (*Madoqua kirkii*), gerenuk (*Litocranius walleri*), common eland (*Taurotragus oryx*), giant forest hog (*Hylochoerus meinertzhageni*), plains zebra (*Equus quagga*), East African oryx (*Oryx beisa*), and the Maasai giraffe (*Giraffa camelopardalis tippelskirchii*). Carnivores in the TCA include cheetah (*Acinonyx jubatus*), African wild dog (*Lycaon pictus*), lion (*Panthera leo*), and leopard (*Panthera pardus*), and spotted hyena (*Crocuta crocuta*).

Rainfall in the TCA is bimodal, ranging between 200 and 700 mm per annum^52^. Normal daily temperatures range between 20 and 30 °C^52^. The TCA has an undulating topography with dotted hills like the Yatta Plateau and Taita hills. The vegetation is dominated by *Acacia–Commiphora* bushlands and thickets, with densities of trees and shrubs varying from grasslands to dense shrublands and riparian forests.

### Transport infrastructure

The TCA is bisected by the Standard Gauge Railway (SGR) which runs from the coastal city of Mombasa to Naivasha through the interior cities and towns in Kenya, including Nairobi. Also bisecting the TCA is an old Meter Gauge Railway (MGR) that runs parallel to the (SGR) from Mombasa to the interior cities. Additionally, major roads transverse the protected area including the Mombasa-Nairobi highway, which parallels the SGR and MGR, the Voi-Taveta highway, and the Manyani-Malindi highway (Fig. 1b). Of the transportation infrastructure in the TCA, the recently built SGR (construction started March 2015 and operational June 2017) is the primary interest of this study. The SGR, unlike the MGR and adjacent highways, was designed to facilitate wildlife movement, specifically continual migration, and dispersal of wildlife within the TCA landscape. This was achieved initially by mapping traditional paths used by elephants, a flagship species in the TCA, then designing and constructing bridges along six crossing points, namely the Maungu, Bachuma, Ndara, Kenani, Manyani and Kanga as wildlife underpasses or passages. In addition, a bridge was constructed along the SGR where it crosses the Tsavo River. These bridges have varied lengths and heights, with some ranging up to 2 km in length (Table S1). Further, several culverts were constructed for drainage purposes and to facilitate wildlife crossings. As part of the structural design, the SGR is constructed on raised ground adjacent to bridges and culverts, creating steep embankments on either side of the railway track; there are no wildlife overpasses along the SGR. On either side of the embarkment is an electric fence, erected January 2018, to funnel wildlife to the various underpasses to reduce the risk of trains colliding with wildlife and to minimize the risk of injury to wildlife due to falling from the embankments.

### Underpass use

From June 2016 to October 2019, data on the use of SGR underpasses by medium- to large-sized mammals were collected by direct and indirect observation along two sections of the SGR within the TCA. The first section was Voi to Bachuma (VB) which traverses Tsavo East National Park and the community ranches (Fig. S1b) and Voi to Mtito-Andei (VM) which traverses Tsavo East and West National Parks (Fig. S1a). VB was visited 164 times over a 4-year period, while VM was visited 167 times during the same period (June 2016–October 2019), approximately three-four visits per month for both sections. The visits were carried out by driving a vehicle the entire length of the SGR at a speed of 40 km per hour—the maximum speed allowed inside the park—to enable us to reach underpasses and embankment. During each visit 3 observers and a driver stopped at underpasses, and embarkments to inspect any direct sightings of wildlife and livestock crossings and indirect signs. Direct sightings included animals being observed crossing the SGR at any point during the surveys and indirect evidence of underpass use included footprints, feces, pellets, and droppings on the underpasses.

Mammalian species’ tracks were identified using track keys^53^. All the underpasses had open soil substrate without vegetation, allowing for track identification. Generally, tracks were divided into hooved and pawed impressions. Pawed animal tracks were identified using the size of the track and presence or absence of claws. For similarly sized animals, the shape of the paws and proportion of the interdigital pad to the paws and other distinguishing characteristics were used to differentiate species^53^. Unclassified paw tracks were pooled as carnivores. Hoofed tracks were classified to into species or appropriate taxon based on the presence and absence of cloven hooves, size of the hooves, number of toes, and the shape of the hoofs. Hoofed tracks were recorded as antelope if they could not be identified to species or genus level. If tracks in the underpass were not clearly identifiable, they were followed to where the substrate could allow identification. To avoid double counts on subsequent days, footprints were erased with a feather duster so as to prevent recount^54^. Scats were only recorded if they were fresh^53^. To avoid double counts scats were marked with white chalk.

**Figure 2:**
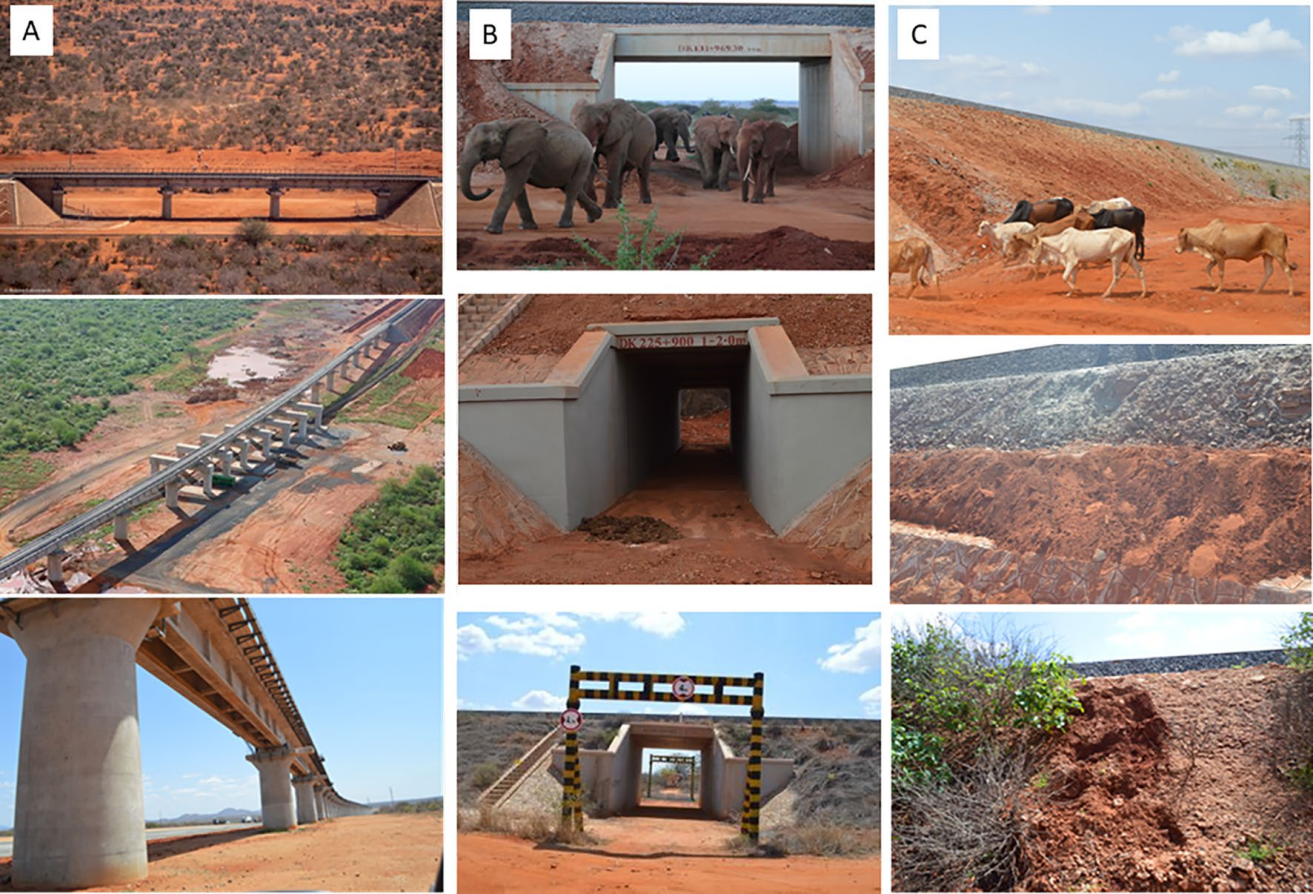
Wildlife crossings were classified as bridges (**A**), culverts (**B**), and embankments (**C**) along the standard gauge railway in Tsavo National Parks, Kenya.

For this study, underpasses were mapped using center GPS locations (Fig. S1) Wildlife crossings were classified into three categories: bridges, culverts, and embankments (Fig. 2A–C). Bridges were defined as raised sections of the railway supported by piers and abutments and spanning more than 6 m in length and more than 6.5 m high. A culvert was a tunnel structure built to allow water and wildlife to pass and are usually embedded in the soil and are less than 6 m in length with varying height. Embankments were compacted earth material that raised the grade line of a highway or railway. We monitored wildlife crossings in 14 bridges, 58 culverts and 69 embankments in two sections of the SGR namely, park-ranch interface (VB), and park-park interface (VM) (Fig. S1).

### Species or taxon abundance

Data on the abundance of medium-sized to large-sized mammals (species with a mass greater than ca. 2 kg, see^55^) near TCA roads were collected from monthly road counts taken from July 2008 to July 2015. Vehicle road counts were conducted by driving along road transects at a fixed speed of 20 km-hr to enhance both detection and increase greater road distance coverage. While driving, we stopped to take a count, identify the species when a wildlife species was sighted on either side of the road up to 250 m. Distance to the animal or group was measured with a Bushnell Scout DX 1000 Laser Rangefinder. The road count was carried out along three major road transects: Voi-Bachuma-Satao (145 km), Aruba-Sala (109 km) and Voi-Buffalo Wallows-Manyani (128 km) within Tsavo East National Park. Road counts started at 6.00 a.m. and ended when the transect distance was achieved. All mammals, from dik-dik to elephant were recorded during the count, locations were mapped, and behavior was recorded. These data were used to derive the expected frequency of mammal use of the underpasses from the proportion of their proximate abundance estimates along roads.

### Ecological factors

Ecological variables associated with underpasses (i.e., Normalized Difference Vegetation Index (NDVI), and proximity to water sources) were extracted from remotely sensed data and from drainage maps of the Tsavo ecosystem, respectively. NDVI products derived from SPOT VGT were downloaded from ESA (European Space Agency) (https://earth.esa.int/web/guest/data-access/browse-data-products). The NDVI products downloaded were for period January 2016 to December 2019 covering the study duration to obtain a single averaged product per month associated with each underpass.

The proximity of SGR underpasses to water sources were obtained from drainage (natural streams and rivers) of the Tsavo East and West digitized from 1:250,000 toposheets including Voi SA-37-14, Kilifi SA-37-15, Garsen SA-37-1, and Kibwezi SA-37-10. Additional data on locations of water tanks, boreholes, dams, troughs, and pans for the Tsavo East and Tsavo West National parks were obtained using Garmin GPS (GPSMAP 64). Straight-line distances between underpasses and the nearest water sources were measured in ArcGIS Toolkit.

### Underpass type, size, and infrastructure

Data on infrastructure variables including underpass dimensions (width, length, and height), proximity of underpass to roads, and presence of a functional electric fence along the embankments adjacent the underpasses were obtained. The length and width measurements for both culverts and bridges were provided by the China Road and Bridge Company (the company in charge of building the SGR) and this information is labelled on some of the underpasses. The major roads were digitized from 1:250,000 toposheets including Voi SA-37-14, Kilifi SA-37-15, Garsen SA-37-11, and Kibwezi SA-37-10. We calculated the distance of each unique underpass point to the nearest highway road. Distances were recorded to the nearest kilometer.

### Statistical analyses

To answer the question of whether some species are more likely than others to use underpasses, we employed a chi-square analysis using observed data on the frequency of underpass use by the top 20 most sighted species (Table S2). We calculated the expected frequency of underpass crossing by each wildlife species using wildlife species abundance data along roads. Computation of expected frequencies and chi-square analyses were conducted using R software for statistical computing^56^.

To determine whether fencing, underpass type and dimensions, NDVI, proximity to rivers and other water sources, and roads affected crossing by a selected species or taxa, we modelled covariate effects with a generalized linear mixed effect model (GLMM) framework using a logit link function and a binomial error structure. Underpass ID was employed as random effect. As independent variables, we used the presence or absence of crossings along wildlife passages by selected species or taxonomic groups which had 20 or more sightings from routine monitoring of the SGR. These species include, savannah elephant (*Loxodonta Africana*), African buffalo (*Syncerus caffer*), plain’s zebra (*Equus burchellii*), yellow baboon (*Papio cynocephalus*), Kirk’s dik-dik (*Madoqua kirkii*), lion (*Panthera leo*), leopard (*Panthera pardus*), spotted hyena (*Crocuta crocuta*), African civet (*Civettictis civetta)*, impala (*Aepyceros melampus*), waterbuck (*Kobus ellipsiprymnus*)*,* and lesser kudu (*Tragelaphus imberbis*). We also grouped species into the following categories: antelope, carnivore, or mongoose and livestock or wildlife and used these as dependent variables as well.

To address the question of whether livestock presence hindered or enhanced the use of underpasses by wildlife, livestock presence or absence was used as an explanatory variable and each of the wildlife species or taxonomic groups were used as dependent variables. In addition, when lion was used as dependent variable, plains zebra, African buffalo and livestock were included as independent variables because these are preferred prey species^57–61^. When leopard, and spotted hyena were employed as dependent variables, antelope, zebra, and livestock were included as independent variables, as these are these are some of their preferred prey species^62–64^. For all carnivore species (identified to species or not), we used antelopes as an independent variable.

GLMMs were performed using the glmmTMB package^65^. We used AIC model selection to distinguish among a set of possible models describing the relationship between infrastructure design and ecological factors and mammalian use of SGR underpasses in the TCA. For each model we included a comprehensive list of all independent variables the best set of covariate influencing the likelihood of using the underpass was evaluated using AIC in the MuMIn package^66^. All the software packages are part of the R software for statistical computing^56^.

## Results

### Differential utilization of the SGR underpass by various wildlife species

Thirty-three species of medium- to large-sized mammals were observed using the SGR underpasses, including unidentified species grouped in general categories of carnivore, mongoose, and antelope. Livestock (e.g., cattle, goats, sheep, donkeys, and camels) were also frequently observed to use the SGR underpasses (Tables S2, 1). The top five wildlife species that utilized the underpass bridges (percent of observations) were elephant (30.48%), plains zebra (20.23%), baboon (12.35%), buffalo (7.58%), and dik-dik (7.71%) (Table S2). Similarly, the top species using culverts were elephant (3.63%) and baboon (2.74%). Livestock used the culverts more than any wildlife species (11.16%). Hyena was the top identified carnivore utilizing the underpasses, but the top five carnivores using the underpasses frequently, includes leopard, lion, black-backed Jackal, and civet (Table 1).

**Table 1:** The frequency and percentage use of underpass by medium- to large-sized mammals in the Tsavo Conservation Area ranked by observed bridge crossings.

Species/taxon	Scientific name or family/order name	Bridge		Culvert	
		Observed count	Percent of total count	Observed count	Percent of total count
Savannah elephant	*Loxodonta africana*	708	30.48	348	3.63
Plain’s zebra	*Equus quagga*	470	20.23	97	1.01
Livestock	Bovidae	389	16.75	1070	11.16
Yellow baboon	*Papio cynocephalus*	287	12.35	263	2.74
African buffalo	*Syncerus caffer*	176	7.58	135	1.41
Kirk's dik-dik	*Madoqua kirkii*	179	7.71	103	1.07
Spotted hyena	*Crocuta crocuta*	129	5.55	117	1.22
Mongoose	Herpestidae	76	3.27	164	1.71
Impala	*Aepyceros melampus*	57	2.45	8	0.08
Antelope	Bovidae	55	2.37	46	0.48
Carnivore	Carnivora	50	2.15	67	0.70
Leopard	*Panthera pardus*	19	0.82	40	0.42
African civet	*Civettictis civetta*	36	1.55	2	0.02
Waterbuck	*Kobus ellipsiprymnus*	17	0.73	5	0.05
Lesser kudu	*Tragelaphus imberbis*	20	0.86	14	0.15
Lion	*Panthera leo*	13	0.56	9	0.09
Black-backed jackal	*Canis mesomelas*	8	0.34	13	0.14
Vervet monkey	*Chlorocebus pygerythrus*	21	0.90	0	0.00
Common warthog	*Phacochoerus africanus*	5	0.22	11	0.11
Grant’s gazelle	*Nanger granti*	8	0.34	4	0.04

**Figure 3:**
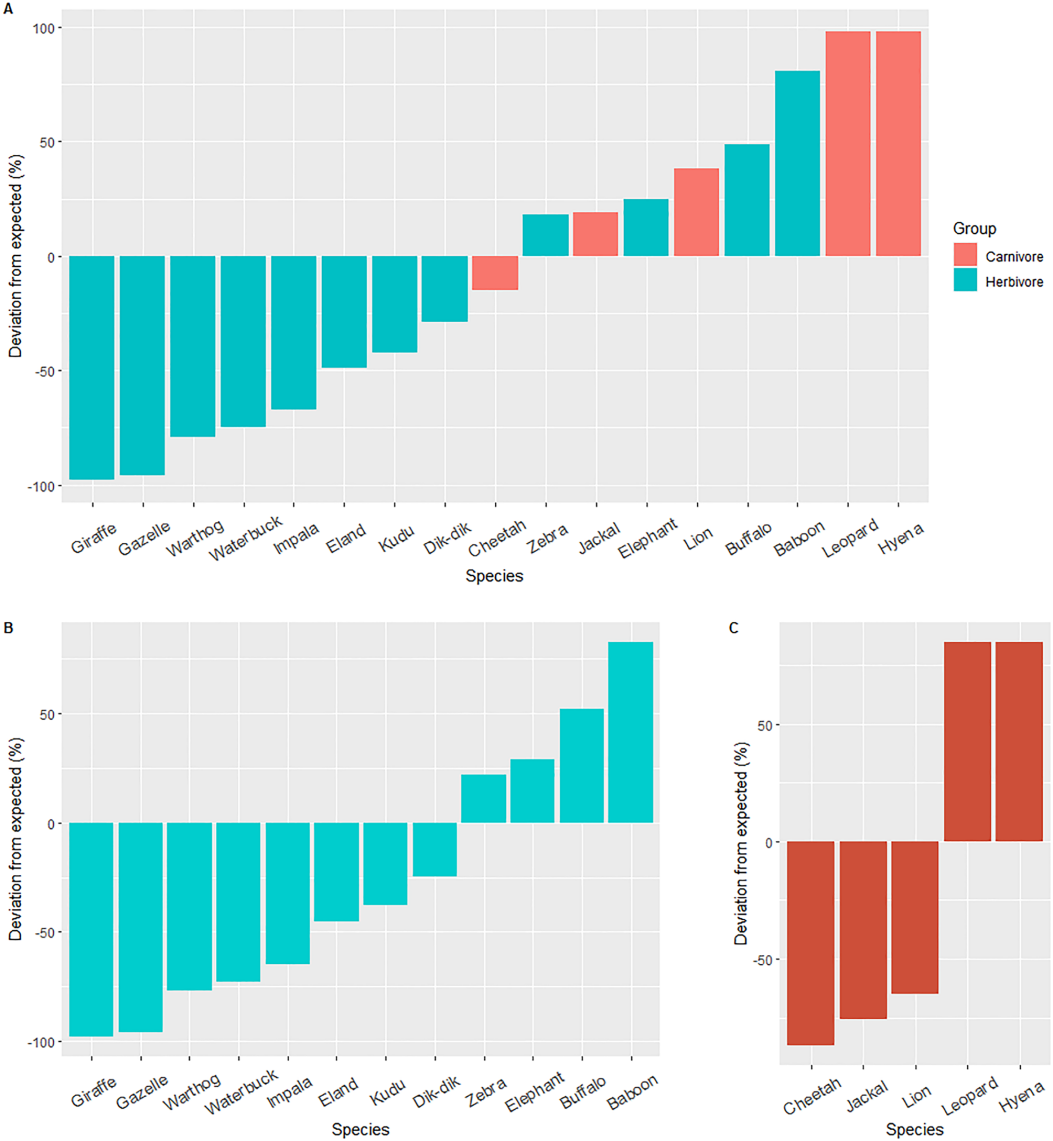
The percent deviation of SGR underpass utilization by medium- to large-sized mammals when herbivores and carnivores are simultaneously considered (**A**) and when herbivores (**B**) and carnivores (**C**) are independently considered. The expected frequency was calculated based on the frequency of their sighting on TCA roads.

The most abundant wildlife species sighted from monthly road counts over a 7-year period were elephant, Grant’s gazelle, Kirk’s dik-dik, plains zebra and impala. Black-backed jackal and lion were the most sighted carnivores, but species such as hyena and leopard were less sighted during road counts (Tables S3, 2). Some species frequently sighted among the top 20 during road counts were also observed to be among the most frequent users of the SGR underpasses. These include the savannah elephant, African buffalo, Kirk’s dik-dik, impala and yellow baboon. However, there were also some species observed frequently during road counts that were observed infrequently using the SGR underpasses. These infrequent underpasses users were Maasai giraffe, Coke's hartebeest, common eland, common hippopotamus, and Grant’s gazelle (Tables S2, S3). Generally, the frequency of underpass use by different species was not dependent on their corresponding monthly road count frequencies (*χ*^2^_*17*_ = 45,698, P < 0.0001; Fig. 3a). This was also the case when we separately tested whether underpass use by carnivores and herbivore species was expected based on their abundance from road sighting (herbivores: *χ*^2^_*11*_ = 7265.7, P < 0.0001, Fig. 3b; carnivores: *χ*^2^_*4*_ = 3429.4, P < 0.0001, Fig. 3c).

**Table 2:** The influence of electric fencing on the percentage use of SGR underpasses by wildlife in the TCA.

Taxon or species identity		Underpass (%)		Embankment (%)	
Species/taxon common name	Scientific name or family/order name	Unfenced	Fenced	Unfenced	Fenced
Savannah Elephant	*Loxodonta africana*	10.28	7.30	4.89	0.09
Yellow baboon	*Papio cynocephalus*	2.42	7.05	0.00	0.00
Plain’s zebra	*Equus quagga*	3.69	5.94	0.64	0.00
African buffalo	*Syncerus caffer*	1.60	3.73	0.72	0.00
Kirk’s Dik-dik	*Madoqua kirkii*	1.22	3.64	0.00	0.00
Spotted hyena	*Crocuta crocuta*	1.14	3.09	0.05	0.00
Impala	*Aepyceros melampus*	0.30	0.81	0.15	0.00
Leopard	*Panthera pardus*	0.53	0.46	0.03	0.02
Lesser kudu	*Tragelaphus imberbis*	0.21	0.37	0.00	0.00
Vervet monkey	*Chlorocebus pygerythrus*	0.03	0.34	0.00	0.00
African civet	*Civettictis civetta*	0.32	0.32	0.00	0.00
Lions	*Panthera leo*	0.10	0.28	0.07	0.00
Common warthog	*Phacochoerus africanus*	0.08	0.19	0.07	0.00
Cape hare	*Lepus capensis*	0.02	0.14	0.02	0.00
Crested porcupine	*Hystrix cristata*	0.00	0.11	0.00	0.00
Waterbuck	*Kobus ellipsiprymnus*	0.27	0.09	0.21	0.00
Black-backed jackal	*Canis mesomelas*	0.27	0.07	0.02	0.00
Caracal	*Caracal caracal*	0.13	0.05	0.00	0.00
Livestock	Bovidae	8.06	16.88	0.00	0.00
Antelopes	Bovidae	0.22	1.54	0.00	0.00
Carnivore	Carnivora	0.67	1.33	0.00	0.00
Mongoose	Herpestidae	0.27	3.94	0.00	0.00

### Effect of fencing on underpass and embankment utilization by wildlife and livestock

Many species used embankments to cross the SGR, but most of them stopped using embankments following the installation of an electric fence (median of times crossed before = 4 and median of times crossed after = 0, V = 91, P = 0.0016, Wilcoxon test for matched pairs), reducing the risk of wildlife mortalities from train-wildlife collisions. The exceptions were elephant, and leopard, but even for these, electric fencing dramatically reduced their use of embankments. Electric fencing increased underpass use by most species, except for elephant, black-backed jackal, caracal, and waterbuck (Tables 2, S4) where fencing reduced the rate of underpass usage (Table 3) and leopards and civets, for which fencing exhibited no discernable effect.

**Table 3:** Coefficients from the best of several models for each mammalian species or group based on AIC showing the influence of infrastructure design and ecological factors on Standard Gauge Railway underpasses use in Tsavo Conservation Area.

Dependent variables	Independent variables												
	Intercept	Livestock	Distance to road	Electric fencing	Underpass height	NDVI	Underpass (culvert vs bridge)	Distance to water-source	Underpass width	Antelope present	Zebra present	Buffalo present	Wildlife present
Savannah elephant	− 4.033***	NA	NA	− 0.459***	0.512***	NA	− 1.261**	− 0.059+	NA				
African buffalo	− 9.025***	− 2.075***	1.29**	1.172***	0.643***	1.156***	NA	− 0.224***	0.0018***				
Plain’s zebra	− 9.425***	− 0.899**	− 5.711+	1.322***	0.682*	NA	− 2.058	0.283*	NA				
Yellow baboon	− 7.116***	− 0.256	− 2.899**	1.464***	0.755***	− 3.139***	NA	NA	NA				
Kirk’s dik-dik	− 13.493***	NA	NA	1.757***	1.342***	− 7.173***	NA	NA	NA				
African civet	− 13.64***	NA	NA	NA	0.930***	NA	NA	NA	NA				
Impala	− 5.808***	NA	NA	1.241***	NA	− 2.316	− 4.013***	NA	NA				
Lesser Kudu	− 14.542***	NA	NA	0.938*	1.164**	− 9.165**	NA	NA	NA				
Black-backed Jackal	− 8.609***	− 18.357	NA	− 1.234*	0.373*	NA	NA	NA	NA				
Lion	− 9.235***	1.085+	NA	0.826+	0.307*	NA	NA	NA	NA		1.334*	1.473*	
Spotted hyena	− 10.777***	NA	NA	1.012***	0.88***	2.316***	NA	NA	− 0.0015	0.805**	NA		
Leopard	− 15.546***	− 17.256	NA	NA	1.343***	NA	1.86	0.172	− 0.0075	1.537*			
Mongoose	− 8.122***	− 0.544*	− 2.356+	3.124***	0.685***	− 4.580***	NA	− 0.163*	NA				
Antelope	− 9.418***	− 1.179**	NA	1.501***	0.949***	− 1.730***	NA	NA	NA				
Carnivores	− 9.508***	NA	NA	0.51*	0.614***	NA	NA	NA	NA	1.314***			
Livestock	1.804		− 14.658**	1.145***	NA	1.656***	NA	− 1.189***	NA				− 0.256*
Wildlife	− 4.905***	− 0.366***	NA	0.779***	0.723***	− 0.518+	− 0.975+	NA	NA				

### Effect of infrastructure, underpass type and dimensions on wildlife and livestock utilization of underpasses

Among the underpass design factors, height was a more important factor than either type (bridge or culvert) or width because it was selected in nearly all the models whereas bridge type or width were selected in 5 and 3 models respectively (Table 3). Specifically, there was a positive relationship between underpass height and the probability of underpass use by mammals (see coefficients in Table 3). Underpass type was important for some mammalian species, but its effect was weak. Generally, culverts were used to a lesser extent relative to bridges and this effect was stronger for elephants (Table 3).

The distance of the underpass to the Nairobi Mombasa highway had varied effects on different species. Baboons and livestock preferred to use underpasses closer to the highway whereas buffalo and antelopes preferred to use underpasses farthest from highways. Proximity of the underpass to the highway did not influence their crossing by most carnivores considered in the analyses (Table 3).

### Effects of ecological factors on wildlife and livestock utilization of underpasses

Underpasses located in areas with higher NDVI were more likely to be used by buffalo, livestock and hyenas and these species were also more likely to use underpasses in proximity to water sources. In contrast, baboon, dik-dik and antelope avoided to utilize underpasses with high NDVI (Table 3). However, the plains zebra preferred underpasses farthest from perennial water sources (Table 3).

Livestock presence or use of underpasses reduce the likelihood of underpass use by most wildlife species except baboons and most carnivore species (Table 3). The utilization of underpasses by lion, leopard, hyena, and unclassified carnivore species was influenced by presence of their prey species. Lions were more likely to use underpasses where zebra buffalo and livestock (i.e., their key prey species) were present. Leopard, hyena, and other carnivores used underpasses where their prey species, antelope, were also present (Table 3).

Probability values indicated by asterisk (*** < 0.001, ** < 0.01, * < 0.05, +  < 0.1), NA indicates independent variable dropped during model selection. For full list of all models and their AIC, see supplementary model selection excel workbook.

## Discussion

We determined that many wildlife species use the SGR underpasses, but species such as the Maasai giraffe, common warthog, impala, Coke's hartebeest, common eland, and Grant’s gazelle had a lower propensity to use underpasses, whereas most carnivores and baboons had a higher propensity to use underpasses than expected. The rarity of underpass use by giraffe, and the strong positive correlation between underpass use by most wildlife species and underpass height observed, highlights the limitation giraffes face in using underpasses. Giraffe with their long neck and legs, have an extended viewing horizon to maintain vigilance^67^, and may even view bridges as obstacles with the small difference between their height and that of the bridges. For example, the average height of giraffe is about 5.5 m for males and 4.3 m for females^68^ and the modal height of bridges in this study is 6 m (see “Discussion” on the influence of underpass height below), but most culverts are inaccessible to giraffes due to their low heights (3–4 m).

For other species, predator–prey interactions may explain observed less than expected use of the underpasses. Indeed, the use of underpasses by lion, leopard, and hyena, was positively influenced by presence of buffalo, zebra, and antelope, which are their key prey species^59,62,64^. This suggests that predator–prey interactions were important factors in underpass use by the major carnivores in the TCA. These findings are similar to those found for coyotes, *Canis latrans* in California, where coyotes favored underpasses with high presence of their main prey items, rodents and lagomorphs^26^. Mata et al.^69^ concludes that wildlife crossing structures will be less effective for prey if their use is adversely influenced by predator–prey interactions. A similar finding occurred regarding the use of underpasses by southern brown bandicoot (*Isoodonobesulus fusciventer*) and their fox (*Vulpes vulpes*) predator^70^. Such may have been the case for species such as common warthog, impala, grant gazelle, dik-dik, eland, waterbuck, and lesser kudu, which all used underpass less than expected. While at the same time their main predators, leopards, and hyenas, used the underpass more than expected even when potential bias is adjusted by considering cryptic carnivore alone.

It is also important to consider that some wildlife species more readily adapt to human-dominated landscapes. Such species include non-human primates, like chacma baboon (*Papio ursinus*) and tokean macaques (*Macaca tonkeana*)^71–73^, and opportunistic carnivore species, such as spotted hyena^74,75^, leopard^76–79^, African lion^80^ and coyote (*Procyon lotor*)^81,82^. Not surprisingly then, yellow baboons, spotted hyenas, leopards and to a lesser extent lion, used the underpass more than expected.

This study also revealed that electric fencing reduced the rate of SGR crossing by wildlife at the embankments while increasing the use of underpasses. Elephants, however, were an exception to this finding and their use of underpasses after the electric fences were erected was reduced. This suggests that while fencing can help reduce wildlife mortalities from train-wildlife collisions by helping to funnel wildlife through underpasses, electric fencing may also reduce elephant connectivity. Other studies have found that fencing has a funneling effect that directs larger animals toward culverts^46^. Underpasses when combined with fencing have been shown to reduce large mammal–vehicle collisions by 86% on Highway 93 in Montana, United States, while also maintaining wildlife connectivity across roads^22^. The effectiveness of crossing structures is significantly enhanced when combined with fences, and both measures are usually best implemented together^17^.

However, fencing although effective, may reduce overall permeability of landscapes traversed by roads. For example, we observed a reduction in underpass use by elephant and black-backed jackal following the erection of electric fencing along the SGR. Other species that reduced underpass use include the caracal, leopard, and waterbuck. These findings suggest that species with traditional migratory routes or those that defend territories may be adversely affected by electric fencing. For example, elephant families have traditional movement routes^35,83,84^ and if underpass structures do not take this into consideration many elephants may fail to cross the SGR. Its therefore important that the underpass structures take into consideration the traditional elephant routes to ensure their immediate use. It has been observed, for example, that underpasses placed at identified panther (*Felis concolor cory*) crossing points along US I-75 Collier county Florida, using prior knowledge of panther movements, were more likely to be used by panthers^25^. Similarly, deer underpasses placed along US Interstate Highway 84 in Idaho without regard to traditional paths failed, irrespective of addition of fences, to direct deer to those crossings^25,85^. Fencing has been shown to reduce underpass vehicle moose collisions, but also to reduce the use of underpasses in southwestern Sweden along European highway 6^23^. These findings underscore the need for extensive wildlife movement monitoring prior to road construction, so that underpasses can be located along natural wildlife routes.

Black-backed jackals, leopards, caracals and waterbucks are all territorial species^86,87^ that had reduced underpass use following fencing. Our results suggest that fencing along the SGR, like along highways, may impose artificial home range boundaries on some territorial species^88^. This suggest a need to examine more explicitly the impact of road and rail infrastructures on home range displacement and abandonment^89^.This study also revealed that livestock presence reduces the likelihood of underpass use by most wildlife species except baboons and most carnivore species. Several studies report spatial segregation between cattle and wildlife^90–92^. For example, Hibert et al.^91^ found spatial avoidance between cattle and wildlife grazers including elephants in the trans-frontier W Regional Park in Burkina Faso, Benin and Niger. Another study found that elk (*Cervus elaphus nelsoni*), mule deer (*Odocoileus hemionus hemionus*), and cattle which frequently co-occur in the northwestern United States^92^ were spatially segregated and avoided each other. The occurrence of livestock also suggests the presence of humans and many studies have shown wildlife avoidance of human or livestock presence^90,91,93^.

Among the underpass design factors, this study revealed that height is more important than either type of underpass (bridge or culvert) or width in affecting use by wildlife and livestock. Similarly, in Virginia, USA, underpasses with a minimum height of 4 m were successful in facilitating the passage of white-tailed deer (*Odocoileus virginianus*) and other wildlife species^24^. Our result also concurs with a study in Banff National Park, Alberta, Canada where crossing structures that are high, wide, and short in length strongly influence grizzly bear (*Ursus arctos horribilis*) passage^27^. Structural designs are the main determinant in species’ use of wildlife passages along roads and highways. It is thought that animals using an underpass require an unobstructed view of the habitat or horizon on the far side of the underpass to ensure safety^25^. Moreover, some studies^94,95^ have used the openness index ratio, a measure of an animal’s ability to see into the other side of the underpass. It has been suggested that this feature is probably is more important than the exact width and height of the underpass as it integrates both height width and length^96^. Wildlife overpasses which offer a better view of where animals are moving to were utilized more than underpasses by mule deer^20^. Large mammals appear to prefer overpasses compared to small mammal use of underpasses^20,97^. For livestock herded into the park, it is not surprising that height or width were not statistically significant variables influencing their use of the underpasses because they are not making movement decisions independent of their shepherds.

This study also showed that parallel infrastructure had a varied influence on underpass use by wildlife and livestock. Most railways and roads are usually co‐aligned in the same corridor^12^. This creates a challenge for wildlife as they must cross multiple infrastructure impediments when moving from one side to another. Indeed, we observed in this study that buffalo and unclassified antelope preferred underpasses that were farthest from the highway. In contrast, baboons and livestock used underpasses that were near roads. This was not surprising for baboons as they often scavenge human food leftovers^98^. Along the infrastructure corridor which includes the SGR, food leftovers are often thrown out of cars, particularly in areas close to human habitation (Lala, pers observation). This is likely to attract baboons towards roads and their greater use of wildlife corridors near roads^99^. Moreover, baboons’ proclivity for using wildlife corridors near roads can be further explained by their use of roads as an efficient method of travel, where groups of baboons can move faster and in a more directed manner^100^. Livestock use of underpasses closer to roads is likely due to easy access to the protected resources within the national parks. Livestock are frequently illegally grazed and watered within the Tsavo National Parks. Some of the underpasses used by livestock in this study were the ones closest to areas where the national park boundary interfaces human settlements and communities, often where the highway and the SGR are in closest proximity to one another (Lala, pers observation).

Several mammalian species are known to track spatial and seasonal changes in primary productivity, and to use areas that have lush vegetation or regular access to drinking water (measured as changes in NDVI, and water availability respectively)^101–106^. In this study, we observed that underpasses located in areas with higher NDVI and near water sources were more likely to be used by the African buffalo, livestock, and spotted hyenas. Yellow baboons, Kirk’s dik-dik, lesser kudu, mongoose and antelope preferentially used underpasses with low NDVI. Predation or anthropogenic disturbances have been linked to avoidance of optimal habitats and locations with drinking water sources by wildlife^93^. The preferential use of underpasses that are not near greener areas (i.e., lower NDVI) and far from water sources, might suggest this as a strategy to minimize predation. Some ungulate species use open areas, which are less green, and avoid bushy areas, which have higher greenness^107^. These wildlife species are using these underpasses as passage routes, rather than ones close to foraging areas, because they afford greater safety from predators.

This study demonstrates that to ensure the effectiveness of underpasses, it is critical to consider species’ predator–prey interactions, behaviors, and foraging needs in relation to underpass design and location. With sound wildlife corridor placement and design in areas where railways traverse habitats with diverse wildlife, underpass use by all species should increase. This will become increasingly important in mitigating against habitat fragmentation and guaranteeing safe wildlife passage as transportation infrastructure continues to expand and support greater volumes of traffic.

## Conclusion

For the first time in Africa, we studied the effect of SGR underpass designs on a diverse wildlife community of medium-sized and large-size mammals in a tropical arid ecosystem. We demonstrated that the underpasses limit connectivity for the giraffe and may reduce usage by some prey species due to the presence of their predators and livestock. We also demonstrated the differential effect of wildlife fencing along the SGR on the underpass use by the wildlife community, with positive effects for most species except elephants and leopards. Our results indicate that wildlife friendly underpasses require a diversity of designs including overpasses to address the needs of wildlife of diverse body sizes and ecologies and to improve connectivity and reduce railway kill of species across habitats separated by the SGR.

## Supplementary Information

Supplementary Information 1.

Supplementary Information 2.
