# Supplementary material for: Influence of infrastructure, ecology, and underpass-dimensions on multi-year use of Standard Gauge Railway underpasses by mammals in Tsavo, Kenya
Source: Sci Rep. 2022 Apr 5;12:5698. doi: 10.1038/s41598-022-09555-5 (PMC8983743; doi:10.1038/s41598-022-09555-5)
Supplement: Supplementary file 1 — Supplementary Information 1. [file 41598_2022_9555_MOESM1_ESM.docx]

Supplementary Materials


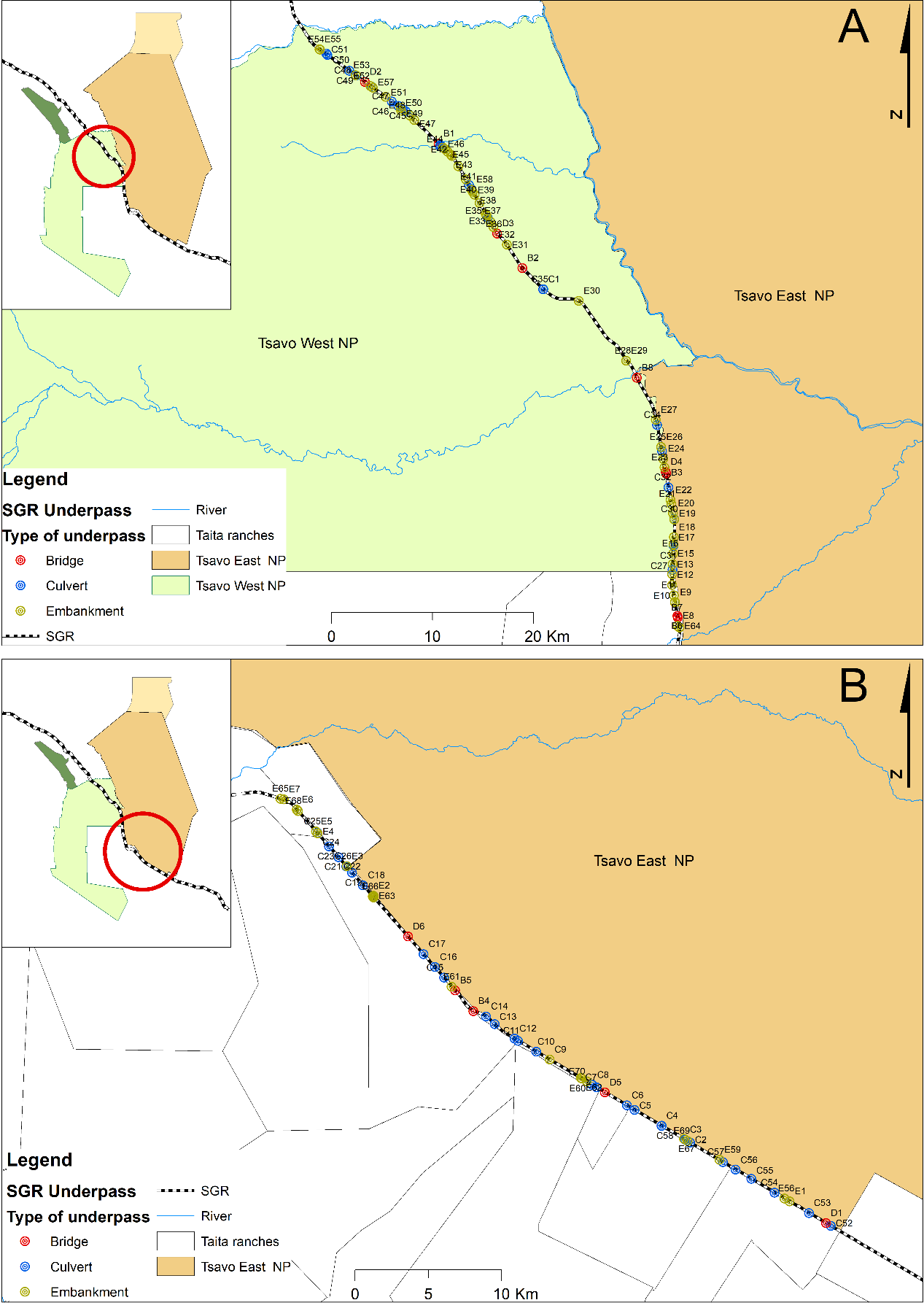


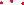


**Figure S1:** Underpass locations between Voi – Mtito-Andei (A) and Voi – Bachuma (B) along the standard gauge railway in Tsavo Conservation Area located in southeastern Kenya (inset). Maps were developed using ArcGIS Software version 10.2.2 (ESRI 2015) and GPS locations of underpasses / embankment used in the mapping were taken by Fredrick Lala.

Table S1. Location, type and dimensions of underpasses examined during this study

| **X- coordinate** | **Y coordinate** | **Underpass ID** | **Type of underpass** | **Road Section** | **Width meters** | **Height meters** | **Size** |
| --- | --- | --- | --- | --- | --- | --- | --- |
| 444273 | 9652304 | C31 | Culvert | VM | 3 | 2 | Medium |
| 417276 | 9695352 | C44 | Culvert | VM | 3 | 2 | Small |
| 443111 | 9661592 | C33 | Culvert | VM | 2 | 2.3 | Small |
| 486140 | 9598800 | C57 | Culvert | VB | 2 | 2.38 | Small |
| 470585 | 9608202 | C13 | Culvert | VB | 3 | 2.4 | Small |
| 442620 | 9664172 | C34 | Culvert | VM | 2 | 2.5 | Small |
| 421115 | 9691604 | C40 | Culvert | VM | 2 | 2.5 | Small |
| 416974 | 9695570 | C45 | Culvert | VM | 2 | 2.5 | Small |
| 416332 | 9696012 | C46 | Culvert | VM | 2 | 2.5 | Small |
| 412121 | 9699034 | C49 | Culvert | VM | 2 | 2.5 | Small |
| 487003 | 9598294 | C56 | Culvert | VB | 2 | 2.58 | Small |
| 461599 | 9617659 | C18 | Culvert | VB | 3 | 3 | Medium |
| 488078 | 9597664 | C55 | Culvert | VB | 6 | 3 | Medium |
| 472172 | 9607062 | C11 | Culvert | VB | 4 | 3.2 | Medium |
| 466513 | 9612090 | C16 | Culvert | VB | 4 | 3.2 | Medium |
| 483905 | 9600126 | C2 | Culvert | VB | 4 | 3.2 | Medium |
| 458404 | 9621328 | C25 | Culvert | VB | 4 | 3.2 | Medium |
| 460495 | 9618961 | C26 | Culvert | VB | 4 | 3.2 | Medium |
| 483566 | 9600336 | C3 | Culvert | VB | 2 | 3.2 | Small |
| 480122 | 9602356 | C5 | Culvert | VB | 3 | 3.3 | Small |
| 431341 | 9677520 | C1 | Culvert | VB | 3 | 3.5 | Small |
| 471936 | 9607214 | C12 | Culvert | VB | 3 | 3.5 | Small |
| 469995 | 9608722 | C14 | Culvert | VB | 3 | 3.5 | Small |
| 460856 | 9618517 | C19 | Culvert | VB | 3 | 3.5 | Small |
| 459942 | 9619567 | C22 | Culvert | VB | 4 | 3.5 | Medium |
| 459283 | 9620313 | C23 | Culvert | VB | 4 | 3.5 | Medium |
| 458514 | 9621231 | C24 | Culvert | VB | 6 | 3.5 | Medium |
| 444334 | 9654896 | C30 | Culvert | VM | 3 | 3.5 | Medium |
| 417426 | 9695248 | C43 | Culvert | VM | 3 | 3.5 | Small |
| 414271 | 9697510 | C47 | Culvert | VM | 3 | 3.5 | Small |
| 412789 | 9698558 | C48 | Culvert | VM | 3 | 3.5 | Small |
| 465736 | 9612968 | C17 | Culvert | VB | 4 | 3.7 | Medium |
| 467890 | 9610500 | B5 | Bridge | VB | 70 | 4 | Large |
| 467142 | 9611366 | C15 | Culvert | VB | 4 | 4 | Medium |
| 460472 | 9618938 | C21 | Culvert | VB | 6 | 4 | Medium |
| 481963 | 9601268 | C4 | Culvert | VB | 6 | 4 | Medium |
| 493493 | 9594462 | C52 | Culvert | VB | 6.8 | 4 | Medium |
| 489667 | 9596716 | C54 | Culvert | VB | 6 | 4 | Medium |
| 481970 | 9601281 | C58 | Culvert | VB | 6 | 4 | Medium |
| 479594 | 9602668 | C6 | Culvert | VB | 6 | 4 | Medium |
| 492016 | 9595328 | C53 | Culvert | VB | 6 | 4.5 | Medium |
| 473414 | 9606324 | C10 | Culvert | VB | 6 | 4.7 | Medium |
| 477244 | 9604060 | C8 | Culvert | VB | 6 | 4.7 | Medium |
| 474334 | 9605790 | C9 | Embankment | VB | 4 | 4.7 | Medium |
| 460485 | 9618949 | C20 | Culvert | VB | 4 | 5 | Medium |
| 477533 | 9603896 | C7 | Culvert | VB | 4 | 5 | Medium |
| 444154 | 9649910 | C27 | Culvert | VM | 6 | 5 | Medium |
| 444274 | 9652288 | C28 | Culvert | VM | 4 | 5 | Medium |
| 444254 | 9652324 | C29 | Culvert | VM | 4 | 5 | Medium |
| 443749 | 9658022 | C32 | Culvert | VM | 4 | 5 | Medium |
| 431348 | 9677528 | C35 | Culvert | VM | 5 | 5 | Medium |
| 424247 | 9687360 | C37 | Culvert | VM | 6 | 5 | Medium |
| 423985 | 9687796 | C38 | Culvert | VM | 6 | 5 | Medium |
| 418131 | 9694624 | C41 | Culvert | VM | 5 | 5 | Medium |
| 417697 | 9695042 | C42 | Culvert | VM | 6 | 5 | Medium |
| 409941 | 9700592 | C50 | Culvert | VM | 4 | 5 | Medium |
| 409835 | 9700722 | C51 | Culvert | VM | 5 | 5 | Medium |
| 493169 | 9594652 | D1 | Bridge | VB | 70 | 5.5 | Large |
| 425844 | 9684556 | C36 | Culvert | VM | 5 | 5.5 | Medium |
| 421151 | 9691706 | C39 | Culvert | VM | 4 | 5.5 | Medium |
| 464682 | 9614172 | D6 | Bridge | VB | 65 | 5.6 | Large |
| 478101 | 9603552 | D5 | Bridge | VB | 70 | 6 | Large |
| 426785 | 9683008 | D3 | Bridge | VM | 70 | 6 | Large |
| 443536 | 9659372 | B3 | Bridge | VM | 20 | 6.5 | Large |
| 443480 | 9659544 | D4 | Bridge | VM | 70 | 6.5 | Large |
| 444661 | 9645378 | B6 | Bridge | VM | 25 | 6.8 | Large |
| 444685 | 9645210 | B7 | Bridge | VM | 60 | 6.8 | Large |
| 413674 | 9697936 | D2 | Bridge | VM | 60 | 7 | Large |
| 469134 | 9609086 | B4 | Bridge | VB | 180 | 7.3 | Large |
| 440619 | 9668820 | B8 | Bridge | VM | 1960 | 9 | Large |
| 429274 | 9679622 | B2 | Bridge | VM | 520 | 10 | Large |
| 420962 | 9691854 | B1 | Bridge | VM | 210 | 12 | Large |

Table S2: Large- and medium-sized mammal sightings in the TCA 2008-2015

| Species/Taxon | Total sightings | Sighting frequency per month |
| --- | --- | --- |
| African elephant | 1677 | 30.491 |
| Grant’s gazelle | 1317 | 23.945 |
| Dik-dik | 1046 | 19.018 |
| Plain’s zebra | 919 | 16.709 |
| Impala | 773 | 14.055 |
| Waterbuck | 497 | 9.036 |
| Gerenuk | 447 | 8.127 |
| Maasai giraffe | 418 | 7.600 |
| Coke's hartebeest | 395 | 7.182 |
| Warthog | 345 | 6.273 |
| Buffalo | 251 | 4.564 |
| Oryx | 186 | 3.382 |
| Kongoni (Hartebeest) | 176 | 3.200 |
| Lesser kudu | 170 | 3.091 |
| Baboon | 117 | 2.127 |
| Hippopotamus | 61 | 1.109 |
| Eland | 54 | 0.982 |
| Black-backed jackal | 31 | 0.564 |
| Vervet monkey | 28 | 0.509 |
| Lion | 24 | 0.436 |
| Common duiker | 19 | 0.345 |
| Banded mongoose | 14 | 0.255 |
| Cheetah | 11 | 0.200 |
| Hirola | 11 | 0.200 |
| Thomson’s gazelle | 9 | 0.164 |
| Topi | 6 | 0.109 |
| Spotted hyena | 4 | 0.073 |
| Klipspringer | 3 | 0.055 |
| Livestock | 2 | 0.036 |
| Bat-eared fox | 2 | 0.036 |
| Rock hyrax | 2 | 0.036 |
| African wild cat | 2 | 0.036 |
| Aardwolf | 1 | 0.018 |
| Bat eared fox | 1 | 0.018 |
| Genet | 1 | 0.018 |
| Grevy’s zebra | 1 | 0.018 |
| Leopard | 1 | 0.018 |
| Reed buck | 1 | 0.018 |
| Rhinoceros | 1 | 0.018 |
| Silver-backed jackal | 1 | 0.018 |

Table S3. The frequency and proportion of time the underpass was used by all large- and medium-sized mammals in the TCA.

| Species/Taxon | Bridge |  | Culvert | | Embankment | | Total | |
| --- | --- | --- | --- | --- | --- | --- | --- | --- |
|  | Count | Ratio | Count | Ratio | Count | Ratio | Count | Ratio |
| Livestock | 389 | 0.1675 | 1070 | 0.1116 | 0 | 0.0000 | 1459 | 0.06242 |
| African elephant | 708 | 0.3048 | 348 | 0.0363 | 303 | 0.0264 | 1359 | 0.05814 |
| Plains zebra | 470 | 0.2023 | 97 | 0.0101 | 39 | 0.0034 | 606 | 0.02593 |
| Yellow baboon | 287 | 0.1235 | 263 | 0.0274 | 0 | 0.0000 | 550 | 0.02353 |
| Buffalo | 176 | 0.0758 | 135 | 0.0141 | 44 | 0.0038 | 355 | 0.01519 |
| Dik-dik | 179 | 0.0771 | 103 | 0.0107 | 0 | 0.0000 | 282 | 0.01207 |
| Spotted hyena | 129 | 0.0555 | 117 | 0.0122 | 3 | 0.0003 | 249 | 0.01065 |
| Mongoose | 76 | 0.0327 | 164 | 0.0171 | 0 | 0.0000 | 240 | 0.01027 |
| Carnivore | 50 | 0.0215 | 67 | 0.0070 | 0 | 0.0000 | 117 | 0.00501 |
| Antelope | 55 | 0.0237 | 46 | 0.0048 | 0 | 0.0000 | 101 | 0.00432 |
| Impala | 57 | 0.0245 | 8 | 0.0008 | 9 | 0.0008 | 74 | 0.00317 |
| Leopard | 19 | 0.0082 | 40 | 0.0042 | 3 | 0.0003 | 62 | 0.00265 |
| Civet | 36 | 0.0155 | 2 | 0.0002 | 0 | 0.0000 | 38 | 0.00163 |
| Waterbuck | 17 | 0.0073 | 5 | 0.0005 | 13 | 0.0011 | 35 | 0.00150 |
| Lesser kudu | 20 | 0.0086 | 14 | 0.0015 | 0 | 0.0000 | 34 | 0.00145 |
| Lion | 13 | 0.0056 | 9 | 0.0009 | 4 | 0.0003 | 26 | 0.00111 |
| Black-backed jackal | 8 | 0.0034 | 13 | 0.0014 | 1 | 0.0001 | 22 | 0.00094 |
| Vervet monkey | 21 | 0.0090 | 0 | 0.0000 | 0 | 0.0000 | 21 | 0.00090 |
| Warthog | 5 | 0.0022 | 11 | 0.0011 | 4 | 0.0003 | 20 | 0.00086 |
| Grant’s gazelle | 8 | 0.0034 | 4 | 0.0004 | 0 | 0.0000 | 12 | 0.00051 |
| Caracal | 3 | 0.0013 | 8 | 0.0008 | 0 | 0.0000 | 11 | 0.00047 |
| Cape hare | 9 | 0.0039 | 0 | 0.0000 | 1 | 0.0001 | 10 | 0.00043 |
| Eland | 0 | 0.0000 | 0 | 0.0000 | 9 | 0.0008 | 9 | 0.00039 |
| Banded mongoose | 1 | 0.0004 | 5 | 0.0005 | 0 | 0.0000 | 6 | 0.00026 |
| Porcupine | 1 | 0.0004 | 5 | 0.0005 | 0 | 0.0000 | 6 | 0.00026 |
| Cheetah | 2 | 0.0009 | 2 | 0.0002 | 0 | 0.0000 | 4 | 0.00017 |
| Genet | 2 | 0.0009 | 2 | 0.0002 | 0 | 0.0000 | 4 | 0.00017 |
| Grevy’s zebra | 1 | 0.0004 | 0 | 0.0000 | 3 | 0.0003 | 4 | 0.00017 |
| Hippopotamus | 3 | 0.0013 | 1 | 0.0001 | 0 | 0.0000 | 4 | 0.00017 |
| Wild dog | 0 | 0.0000 | 3 | 0.0003 | 0 | 0.0000 | 3 | 0.00013 |
| Aardwolf | 1 | 0.0004 | 1 | 0.0001 | 0 | 0.0000 | 2 | 0.00009 |
| Slender mongoose | 2 | 0.0009 | 0 | 0.0000 | 0 | 0.0000 | 2 | 0.00009 |
| Small spotted genet | 1 | 0.0004 | 1 | 0.0001 | 0 | 0.0000 | 2 | 0.00009 |
| Maasai giraffe | 0 | 0.0000 | 2 | 0.0002 | 0 | 0.0000 | 2 | 0.00009 |
| Honey badger | 0 | 0.0000 | 1 | 0.0001 | 0 | 0.0000 | 1 | 0.00004 |
| African wild cat | 0 | 0.0000 | 1 | 0.0001 | 0 | 0.0000 | 1 | 0.00004 |
| Bush baby | 0 | 0.0000 | 1 | 0.0001 | 0 | 0.0000 | 1 | 0.00004 |
| Common duiker | 1 | 0.0004 | 0 | 0.0000 | 0 | 0.0000 | 1 | 0.00004 |

Table S4. The influence of electric fencing on the use of SGR underpasses by wildlife in the TCA.

| Type of underpass | Underpass | | Embankment | | Underpass | | Embankment | |
| --- | --- | --- | --- | --- | --- | --- | --- | --- |
| Fencing Present | N | Y | N | Y | N | Y | N | Y |
| Livestock | 504 | 955 | 0 | 0 | 0.0806 | 0.1688 | 0.0000 | 0.0000 |
| African elephant | 643 | 413 | 298 | 5 | 0.1028 | 0.0730 | 0.0489 | 0.0009 |
| Baboon | 151 | 399 | 0 | 0 | 0.0242 | 0.0705 | 0.0000 | 0.0000 |
| Plain’s zebra | 231 | 336 | 39 | 0 | 0.0369 | 0.0594 | 0.0064 | 0.0000 |
| Mongoose | 17 | 223 | 0 | 0 | 0.0027 | 0.0394 | 0.0000 | 0.0000 |
| Buffalo | 100 | 211 | 44 | 0 | 0.0160 | 0.0373 | 0.0072 | 0.0000 |
| Dik-dik | 76 | 206 | 0 | 0 | 0.0122 | 0.0364 | 0.0000 | 0.0000 |
| Spotted hyena | 71 | 175 | 3 | 0 | 0.0114 | 0.0309 | 0.0005 | 0.0000 |
| Antelope | 14 | 87 | 0 | 0 | 0.0022 | 0.0154 | 0.0000 | 0.0000 |
| Carnivore | 42 | 75 | 0 | 0 | 0.0067 | 0.0133 | 0.0000 | 0.0000 |
| Impala | 19 | 46 | 9 | 0 | 0.0030 | 0.0081 | 0.0015 | 0.0000 |
| Leopard | 33 | 26 | 2 | 1 | 0.0053 | 0.0046 | 0.0003 | 0.0002 |
| Lesser kudu | 13 | 21 | 0 | 0 | 0.0021 | 0.0037 | 0.0000 | 0.0000 |
| Vervet monkey | 2 | 19 | 0 | 0 | 0.0003 | 0.0034 | 0.0000 | 0.0000 |
| Civet | 20 | 18 | 0 | 0 | 0.0032 | 0.0032 | 0.0000 | 0.0000 |
| Lion | 6 | 16 | 4 | 0 | 0.0010 | 0.0028 | 0.0007 | 0.0000 |
| Warthog | 5 | 11 | 4 | 0 | 0.0008 | 0.0019 | 0.0007 | 0.0000 |
| Cape hare | 1 | 8 | 1 | 0 | 0.0002 | 0.0014 | 0.0002 | 0.0000 |
| Porcupine | 0 | 6 | 0 | 0 | 0.0000 | 0.0011 | 0.0000 | 0.0000 |
| Waterbuck | 17 | 5 | 13 | 0 | 0.0027 | 0.0009 | 0.0021 | 0.0000 |
| Black-backed Jackal | 17 | 4 | 1 | 0 | 0.0027 | 0.0007 | 0.0002 | 0.0000 |
| Caracal | 8 | 3 | 0 | 0 | 0.0013 | 0.0005 | 0.0000 | 0.0000 |
| Wild dog | 1 | 2 | 0 | 0 | 0.0002 | 0.0004 | 0.0000 | 0.0000 |
| Hippopotamus | 2 | 2 | 0 | 0 | 0.0003 | 0.0004 | 0.0000 | 0.0000 |
| Honey badger | 0 | 1 | 0 | 0 | 0.0000 | 0.0002 | 0.0000 | 0.0000 |
| Slender mongoose | 1 | 1 | 0 | 0 | 0.0002 | 0.0002 | 0.0000 | 0.0000 |
| African wild cat | 0 | 1 | 0 | 0 | 0.0000 | 0.0002 | 0.0000 | 0.0000 |
| Grevy’s zebra | 0 | 1 | 3 | 0 | 0.0000 | 0.0002 | 0.0005 | 0.0000 |
| Hedgehog | 0 | 1 | 0 | 0 | 0.0000 | 0.0002 | 0.0000 | 0.0000 |
| Aardwolf | 2 | 0 | 0 | 0 | 0.0003 | 0.0000 | 0.0000 | 0.0000 |
| Banded mongoose | 6 | 0 | 0 | 0 | 0.0010 | 0.0000 | 0.0000 | 0.0000 |
| Cheetah | 4 | 0 | 0 | 0 | 0.0006 | 0.0000 | 0.0000 | 0.0000 |
| Genet | 4 | 0 | 0 | 0 | 0.0006 | 0.0000 | 0.0000 | 0.0000 |
| Small-spotted genet | 2 | 0 | 0 | 0 | 0.0003 | 0.0000 | 0.0000 | 0.0000 |
| Bush baby | 1 | 0 | 0 | 0 | 0.0002 | 0.0000 | 0.0000 | 0.0000 |
| Common duiker | 1 | 0 | 0 | 0 | 0.0002 | 0.0000 | 0.0000 | 0.0000 |
| Eland | 0 | 0 | 9 | 0 | 0.0000 | 0.0000 | 0.0015 | 0.0000 |
| Grant’s gazelle | 12 | 0 | 0 | 0 | 0.0019 | 0.0000 | 0.0000 | 0.0000 |
| Maasai giraffe | 2 | 0 | 0 | 0 | 0.0003 | 0.0000 | 0.0000 | 0.0000 |
